# An Evaluation Methodology for Interactive Reinforcement Learning with Simulated Users

**DOI:** 10.3390/biomimetics6010013

**Published:** 2021-02-09

**Authors:** Adam Bignold, Francisco Cruz, Richard Dazeley, Peter Vamplew, Cameron Foale

**Affiliations:** 1 School of Engineering, Information Technology and Physical Sciences, Federation University Australia, Mount Helen, VIC 3350, Australia; a.bignold@federation.edu.au (A.B.); p.vamplew@federation.edu.au (P.V.); c.foale@federation.edu.au (C.F.); 2 School of Information Technology, Deakin University, Geelong, VIC 3220, Australia; richard.dazeley@deakin.edu.au; 3 Escuela de Ingeniería, Universidad Central de Chile, Santiago 8330661, Chile

**Keywords:** reinforcement learning, interactive reinforcement learning, reward shaping, methodology for simulated users

## Abstract

Interactive reinforcement learning methods utilise an external information source to evaluate decisions and accelerate learning. Previous work has shown that human advice could significantly improve learning agents’ performance. When evaluating reinforcement learning algorithms, it is common to repeat experiments as parameters are altered or to gain a sufficient sample size. In this regard, to require human interaction every time an experiment is restarted is undesirable, particularly when the expense in doing so can be considerable. Additionally, reusing the same people for the experiment introduces bias, as they will learn the behaviour of the agent and the dynamics of the environment. This paper presents a methodology for evaluating interactive reinforcement learning agents by employing simulated users. Simulated users allow human knowledge, bias, and interaction to be simulated. The use of simulated users allows the development and testing of reinforcement learning agents, and can provide indicative results of agent performance under defined human constraints. While simulated users are no replacement for actual humans, they do offer an affordable and fast alternative for evaluative assisted agents. We introduce a method for performing a preliminary evaluation utilising simulated users to show how performance changes depending on the type of user assisting the agent. Moreover, we describe how human interaction may be simulated, and present an experiment illustrating the applicability of simulating users in evaluating agent performance when assisted by different types of trainers. Experimental results show that the use of this methodology allows for greater insight into the performance of interactive reinforcement learning agents when advised by different users. The use of simulated users with varying characteristics allows for evaluation of the impact of those characteristics on the behaviour of the learning agent.

## 1. Introduction

**Figure 1:**
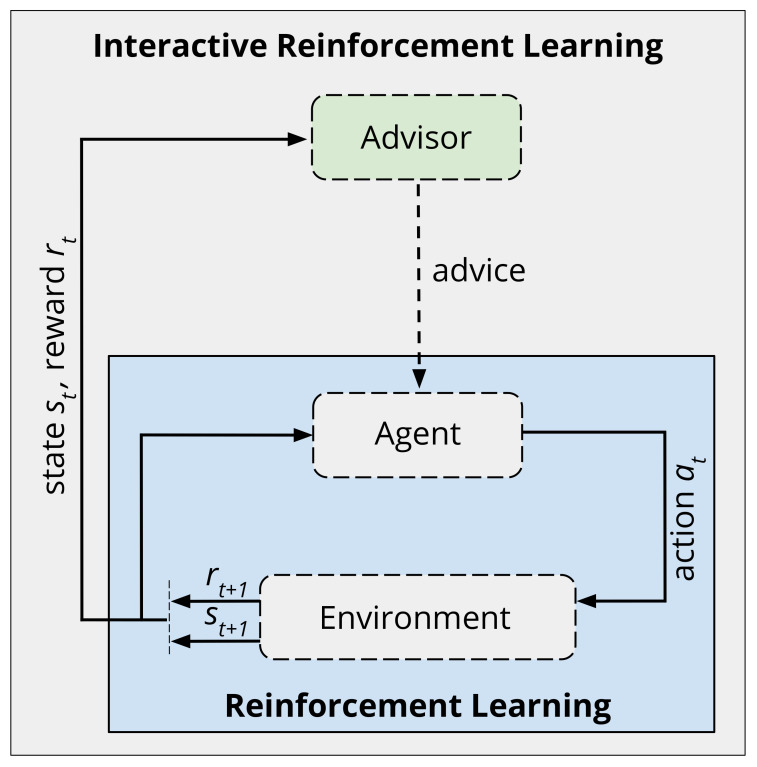
Interactive reinforcement learning approach. An illustration showing the involvement of an advisor and its relation to the traditional reinforcement learning process.

Reinforcement learning (RL) is a machine learning technique that allows artificial intelligence to learn from experience. RL agents attempt to refine their behaviour through interaction with the environment [1]. Using a trial and error approach, an RL agent can observe how performed actions affect the agent’s state and the reward obtained. In Figure 1, the blue box shows the classic RL loop between a learning agent and its environment. In this regard, the sequence of actions an agent chooses to take, given the information it has learned about the problem, is known as the agent’s policy. Ideally, the agent learns the steps that lead to an intended outcome by reinforcing the desired behaviour with a reward value [2].

Initially, RL was used to find solutions to narrow problems such as Tic-tac-toe and Backgammon [3,4]. However, this straightforward and limited framework had difficulties in scaling up to real-world problems [5,6]. Therefore, Kaelbling et al. [7] argued that to solve highly complex problems, RL must give up its *tabula rasa* learning approach and begin to utilise information from outside of the environment.

Assisted reinforcement learning (ARL) techniques utilise information provided by advisors, entities external to the environment, to leverage the learning process. These outside sources may be demonstrations, other agents, past experiences from other domains, or humans providing evaluative or informative advice [8]. Human-sourced information has shown great potential due to its breadth, depth, and availability [9]. ARL agents that interact particularly with humans during operation are known as interactive agents, these agents have shown large improvements over unassisted agents [10,11,12,13].

In interactive reinforcement learning (IntRL), a human is involved in the agent’s learning process [14,15]. Figure 1 shows the IntRL approach with an advisor observing the learning process and providing advice in selected episodes. While the human-in-the-loop approach to learning is one of the interactive agent’s greatest strength, the human can also often be the biggest obstacle [16]. Moreover, human trials are expensive, time-consuming, suffer from issues with repeatability, and acquisition of participants can be difficult. Therefore, during the first stages of development of IntRL agents, we hypothesise that it is much more convenient to simulate human interactions. This would enable rapid development, and save the real human trails for moments when the agent is complete or stable. Nevertheless, the use of simulated users so far has been addressed with no methodology to properly evaluate the underlying IntRL approach.

This paper proposes the use of simulated users for the evaluation of IntRL agents. In this context, a simulated user is an automated user model that reproduces human characteristics [17,18]. While simulated users are no replacement for real human interactions, the use of them is adequate for preliminary evaluation of agents, and during the development stage of new agents, particularly when indicative results are sufficient for assessing agent performance. Additionally, the use of a simulated user allows characteristics such as accuracy to be specifically set, providing insight into how these characteristics alter agent performance. Therefore, we present a methodology for evaluating IntRL agents by employing simulated users that can suitably replicate some characteristics of human interaction. The proposed simulated user methodology can be applied in different contexts such as human-robot collaboration [19,20,21,22], explainable robotic systems [23,24,25], or bioprocess modelling [26], among others.

## 2. Human-Sourced Information

The purpose of the advisor in IntRL is to provide contextually relevant information to the agent. IntRL technique relies on constant interaction from an advisor to achieve maximum potential. Human-sourced information can provide contextually relevant real-time advice; information that can be used to speed up the agent’s learning process and increase performance. The information may be given either by supplementing the reward function [27], altering the policy [28], or adjusting how the agent makes decisions [9]. One aim of IntRL is to make the process of providing advice to an agent as simple as possible, by using methods intuitive to the advice-giver, and by increasing the utility of each piece of advice given to reduce the need for continued interactions. While human advice can be beneficial to an agent [9,11,29], it does come with many challenges, the first of which is the variability of advice-givers and the information they provide.

### 2.1. Characteristics of Human Interactions

Several characteristics of human interaction may impact the quality and applicability of the information humans provide [8,16,30,31]. These characteristics of human-sourced information vary and may include:

- Accuracy: it is a measure of how appropriate information is to the current situation. An information source may be inaccurate due to confusion, poor knowledge, noise, or intentional maliciousness.
- Availability: the information source may not be available all the time or may not respond in the time provided.
- Concept drift: the intentions of the agent and the intentions of the information source may shift over time, such that each time is attempting to work towards a different goal or with a different understanding of the environment.
- Reward bias: advisors may have different teaching styles and prefer to give positive or negative feedback. We classify this as positive reward bias and negative reward bias respectively.
- Cognitive bias: it refers to an advisor’s preconceived thoughts about the nature of the agent and the knowledge they have available to advise the agent in decision making. Advisors are likely to provide advice related to the areas of the domain that they know about and neglect the areas where they know little.
- Knowledge level: an advisor may have little information about all aspects of a problem (breadth), or expert information about a single aspect (depth). Knowledge level may also change over time as the advisor observes the dynamics of the agent or environment.
- Latency: it is a measure of the time taken to retrieve information from the information source. If the latency is too high, then the information may be applied to the wrong state.

These characteristics of human-sourced information can present difficulties when attempting to utilise humans as information sources. Difficulties interacting with people may always be present, therefore, any agent that interacts with a human may experience these issues.

### 2.2. Problems with Human Testing

There are some challenges that need to be considered when using human-sourced information in experiments [32]. The first concern is the expense. Acquiring and employing people for use as an information source can be expensive, more so if domain expertise is required. The expense continues to grow as the number of participants increases. The second concern is the time requirements. It takes some time to acquire, employ, and if necessary, train a participant for a human trial. Additionally, the time required for the human to interact with the agent can significantly reduce the capacity for performing a large number of experiments.

A third concern is a variability between humans acting as information sources, that can lead to a wide disparity in results, depending on their various interaction characteristics. Differences between participants may include teaching style, knowledge, latency, or accuracy [33]. This variability can make it difficult to compare methods reliably. Variability may be mitigated by pretesting participants, while others such as cognitive bias are difficult to identify. All pretests and screening add time and expense to experiments. If variability between participants cannot be reduced, then larger sample sizes are required to achieve statistically reliable results. However, this may also lead to the problem of repeatability of experiments involving humans, the fourth concern. Repeating experiments is essential to gather sufficient sample sizes and identify results with statistical significance but results in increased time and expense. Additionally, participants become increasingly biased as they familiarise themselves with the processes and dynamics of the experiment or become tired or uninterested, making the comparison of methods/agents difficult. Although one solution is to use new participants for each experiment, participants with the required skills can be difficult to source.

While the use of human-sourced information can have a considerable impact on the performance of an RL agent, the number of problems that are inherent to human involvement can make its use difficult. This challenge presents a need for empirical methods for modelling human-sourced information and the characteristics that accompany it [34]. In this regard, simulated users may offer a consistent, reliable, and quantifiable method for replicating human interactions to a degree suitable for providing indicative results.

## 3. Simulated Users

A simulated user is an automated entity designed to replicate the functions of a human user. The purpose is to allow rapid and controlled training and testing. Instead of relying on human assistance, the agent relies on a simulated user whose source of expertise is defined ahead of time. They offer a quantitative method for representing and simulating humans for the evaluation and training of machine learning methods [17,18].

Simulated users are not a replacement for actual humans, however, they do offer a suitable method for gathering indicative results regarding agent performance when assisted. Simulated users are not a method for acquiring new information about the behaviour or a problem, instead, they require an existing solution or collection of pertinent information to be of use to the agent. As such, simulated users are limited to the testing and evaluation of agents on existing problems and not against new domains. Nevertheless, simulated users can address concerns regarding human involvement: expense, time, variability, and repeatability.

A simulated user can be designed to act as a human would in given circumstances. Depending on the complexity and characteristics required, simulated users can, and should, be designed to reflect the qualities of the humans that would be providing assistance to the agent. It is important to exhibit as many as possible of the characteristics of human-sourced information so that accurate evaluations can be performed. For instance, given that human-sourced information is noisy, the simulated users should reflect this. Although assuming that all the range of human characteristics can be completely and accurately replicated is unrealistic, simulated users can provide the necessary functions required to develop and test IntRL systems in place of actual human testing. Furthermore, simulated users can enforce consistency in evaluation, something that humans do not provide necessarily, facilitating the replication and comparability of IntRL techniques.

### 3.1. Applications of Simulated Users

Expert systems face the same challenges as IntRL; expense, time, repeatability, and variability. Expert systems have employed simulated users to evaluate knowledge acquisition methods [32,35,36]. Their use has shown advantages to perform controlled experiments. Empirical studies require repeat experimentation to produce valid results, however, when a user is contributing to an outcome they are learning more about the experiment each iteration, which may bias the results. This problem of variability is compounded further, as users organise their knowledge and priorities quite differently from one another, complicating the method in which controlled studies are performed. Simulated users assist in solving this issue in expert systems, accounting for both variability and scarcity of users [33,36,37]. Compton [33] suggests that the use of simulated user evaluation is possibly the only way to reliably and empirically compare different expert systems.

Spoken dialogue systems (SDS) have also employed simulated users for evaluation [38,39,40]. The development, training, and evaluation of SDS require extensive time interacting with humans, which is an expensive and time-consuming practice. In response, the field adopted the practice of user modelling. User modelling, like simulated users, attempts to design a representation of the intended audience of an SDS [41]. The advantage of adopting user modelling and simulated users to produce training data is that the characteristics of the simulated user can be modified to represent different intended audiences, allowing for better evaluations of the dialogue policies created.

Expert systems and spoken dialogue systems are leading the development of simulated users. Both areas of research suffer from the same issues as IntRL: human testing is expensive and time-consuming, and controlled experiments and comparisons are difficult [33,42]. These fields have shown the benefits that simulated users have for training and evaluation in their respective fields. The development of the simulated user field in these two areas is progressing independently of each other; this implies a lack of structure and terminology about simulated users. Following, we address this by outlining a proposal for different types of simulated users, the principles that they should adhere to, and thoughts on how to reproduce the characteristics of the users they represent.

### 3.2. Evaluation Principles

The success of using a simulated user for evaluation relies on how well it represents the intended audience of the agent. It is important to build a comprehensive and accurate model for the simulated user if reliable indicative results are to be gathered. The strength of a simulated user can be assessed by its adherence to three fundamental principles. These principles are consistency, completeness, and variation. Rieser and Lemon [43] first proposed these principles as a novel way of assessing the ‘naturalness’ of spoken dialogue systems. In this paper, we adopt these principles in IntRL since they are well-suited to the evaluation of simulated users.

- Principle of consistency: states that simulated users should not take actions or provide information that the intended user would not. This principle is constrained to the context of the system being developed and the experimental parameters being tested.
- Principle of completeness: states that simulated users should produce every possible action that the intended user may take. The more complete the action range of the simulated user is the more exhaustive and accurate the evaluation can be.
- Principle of variation: states that simulated users should behave like the users they are modelled from, while not replicating average behaviour completely. To effectively replicate a real user, simulated users must produce outliers and perform unintended actions that, while unlikely, real users may perform.

A simulated user that adheres to these three principles can create a comprehensive system for evaluation. However, while this system is complete in the sense of interaction, it still does not completely reflect the full range of human characteristics [9].

### 3.3. Representing Human-Sourced Information

Representing the characteristics of human-sourced information in simulated users is crucial to perform a suitable indicative evaluation. The intended user of a system is not likely to be perfect, so the simulated user should not be either. By representing the characteristics of human-sourced information a more detailed evaluation can be performed showing how the system handles such factors and how different advisors affect agent performance. Simulated users that model characteristics inherent to human interaction allow a broader evaluation of the agent, as the values of the characteristics can be changed, and the effect measured.

There are three types of models that a simulated user may be based on: probabilistic, heuristic, and stochastic [44]. These methods, or any combination of them, are used to designate how the simulated user is modelled, and how its responses to external signals are decided.

- Probabilistic model: it uses a data-driven approach for representing the intended user of the system [44,45]. The simulated user’s behaviour is defined by probable action choices, probabilities determined by observations of real user behaviour. For example, if users were observed to take action A in 40% of cases, and action B in the remaining, then this would be replicated in the simulated user.
- Heuristic model: it is a deterministic approach for representing the behaviour of a simulated user. Among the most common methods for representing information deterministically are hierarchical patterns [46] and rule sets [47]. Heuristic models are simple to create and maintain, and require little effort to modify. This approach works well when there is little information known about the intended user, but that information is thought to be accurate and reliable.
- Stochastic model: it is an approach used to simulate processes that fluctuate over time, often within a boundary. While it may appear to be like the probabilistic model, stochastic models have a random probability distribution. Examples of stochastic processes include speech and audio signals, data such as temperature and pressure, and medical signals such as EEG and EKG [48]. This approach to modelling is useful for representing complex data and simulating indeterminacy from the intended user.

In Section 5, an illustrative experiment using a simulated user with an heuristic model to represent accuracy and availability is demonstrated. Other more complex simulated users have been used in contexts such as spoken dialogue systems [49] and human-robot scenarios [16]. Particularly in [16], stochastic simulated users have been implemented by previously trained artificial agents. These simulated users learned to solve the task autonomously using a reinforcement learning approach and afterwards are used to train other learning agents with a stochastic level of availability and accuracy. Moreover, other techniques for simulated user implementation may include rules trees and ripple-down rules [50].

## 4. Evaluative Methodology Using Simulated Users in Interactive Reinforcement Learning

The primary contribution of this paper is a methodology for evaluating IntRL agents by employing simulated users. Simulated users offer a method for interacting with an RL agent in place of an actual human, speeding up testing and development, and removing the need for human trials. The use of simulated users as an analogue for human advisors leads to rapid development and for producing indicative results.

The application of simulated users enables a methodical and empirical approach to develop IntRL techniques. This approach is faster and cheaper than using human users, particularly when a broad evaluation of human characteristics is to be tested. Additionally, the use of simulated users does not require human trials or ethics approval, both of which are time-consuming and potentially expensive. Moreover, simulated users provide control over the characteristics of human-sourced information such as accuracy and knowledge level. This control reduces the potential for bias that is often introduced into experiments involving participants. Control of the simulated user model also allows evaluation into the effect of an interaction characteristic on the agent’s performance. For example, how varying levels of interaction frequency affects agent performance. Therefore, this approach is potentially much faster and cheaper than using human users.

Simulated users can also be employed to facilitate the comparison of different IntRL techniques. An issue with the use of humans for testing is that they carry their experiences from past experiments to future experiments. This can result in the human user introducing bias when comparing multiple agents. Unlike humans, simulated users can be reset after each experiment, allowing for objective comparison of IntRL agents and repeatability of experiments.

Evaluations of IntRL employing simulated users can return useful information about how advisors with different characteristics affect the performance of an agent. Simulated users can be modelled to represent a variety of different human users. This ability to model intended users and use them to assess IntRL agents provides insight into how an agent will perform under different conditions. These conditions reflect the characteristics of human-sourced information. For example, these evaluations can demonstrate how an agent performs with increasing amounts of inaccurate information, or how the number of interactions affects performance. Additionally, these assessments of IntRL can be performed very rapidly, and with considerably more control, compared to using human users.

### Proposed Methodology

A general methodology for employing simulated users for the evaluation of IntRL techniques is described here. The purpose of the methodology is to describe a method for using simulated users to interact with an agent so that information can be collected showing how different levels of the human-interaction characteristics (accuracy, availability, etc.) affect the agents learning and performance. The methodology for implementing a simulated user is straightforward, consisting of three phases: construction of the interactive model, implementation of the interactive agent, and evaluation of the interactive approach.

During the first phase, construction of the interactive model, requirements of the analogue are identified, and the user model is created. The model used to represent the simulated user depends on the interaction characteristic being replicated, as some models are better suited to some characteristics than others. Accuracy, for example, may be best represented using a probabilistic model, allowing the level of accuracy to be quickly and easily altered. However, knowledge level may be represented heuristically, as a set of rules can be used to generalise a solution for a large state space. Simulated users may be models from results collected from human trials, generated from datasets, or reverse engineered from environment dynamics. Alternatively, multiple models may be generated to cover a range of possible values for a characteristic. Rather than gaining a baseline accuracy of advice from human trials, instead, a series of simulated users may be generated with varying degrees of accuracy. The results from the set of simulated users can then be used to infer what performance the agent would achieve if assisted by a human of variable accuracy. For instance, if the agent is expecting the user to provide a recommendation for the next action to take, testing how the accuracy of user-sourced advice affects the agents learning may be a possible experiment. In this case, a series of simulated users may be constructed with varying levels of accuracy.

The second phase is the implementation of the interactive agent. The implementation depends entirely on the field of IntRL the agent is being used for, and the role the human is to play in the specific implementation. Whatever the field of RL, the simulated user is used in the same capacity that a real human would be. IntRL employs simulated users to provide evaluation or assisted at the time of learning, while transfer learning uses simulated users to define common behaviours between two domains before learning commences. In this regard, the implementation of the IntRL agent needs to consider elements such as advice interpretation, advice structure, external model, and agent modification. All these elements correspond to processing components and communication links within the assisted reinforcement learning (ARL) framework [8]. Advice interpretation and external model correspond to processing components, they represent how the advice is interpreted (e.g., converted to a supplementing reward signal or to a suggested action) and how the advice is used (e.g., in an immediate or persistent manner), respectively. Advice structure and agent modification correspond to communication links, they represent how the advice is shaped (e.g., as a state-action pair) and how the advice modifies the agent (e.g., reward- or policy-shaping), respectively.

The final phase is the evaluation of the interactive approach. Testing of different agents is performed in the same way as normal human trials, however now the delivery of advice and the human interaction characteristics can be controlled using the simulated user. As the characteristics of the simulated user can be controlled, the bias introduced from real human trials is reduced. The simulated user is reset after each experiment, allowing repeated experiments without the advisor necessarily becoming more familiar with the problem, or introducing its own bias into the results. Additionally, after each set of experiments, the simulated user can be altered to gather data regarding how the change in participant affects the performance of the agent.

After the experiments have been completed the information collected can not only show the agents performance, but this can be compared to the simulated user’s characteristics. This information can allow new insights into IntRL agent behaviour such as how it handles varying degrees of advice accuracy, human availability, concept drift, or knowledge levels. Provided below is an illustrative example of this general methodology. Here, a series of simulated users have been constructed with varying levels of accuracy and availability.

**Figure 2:**
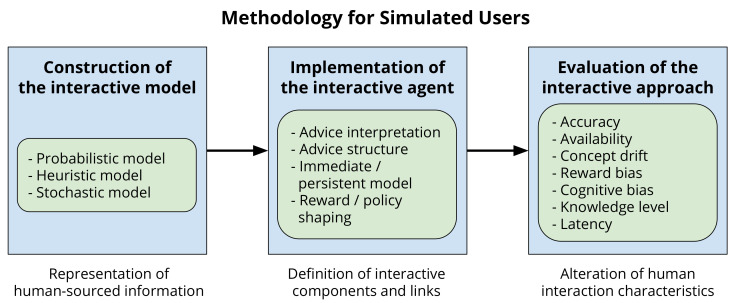
Simulated users methodology. While in the first phase, different representations of human-sourced information can be considered, the second phase considers the definition of processing components and communication links from the assisted reinforcement learning taxonomy. Finally, the third phase includes running experiments to test the approach by altering human interaction characteristics.

Figure 2 outlines the proposed methodology, considering the 3 phases mentioned above and the elements that may be included in each of these phases. In Section 5, we provide an illustrative experiment showing the use of an IntRL agent with a simulated user using the proposed methodology.

## 5. Illustrative Experiment

This section presents an experiment in which an IntRL agent is assisted by a simulated user. The aim of the experiment is to demonstrate the use of a simulated user acting as an analogue for a real user of the system. Furthermore, the experiment evaluates the effect that interaction accuracy and availability have on the performance of a Q-Learning agent in the Mountain Car domain.

### 5.1. Experimental Set-Up

**Figure 3:**
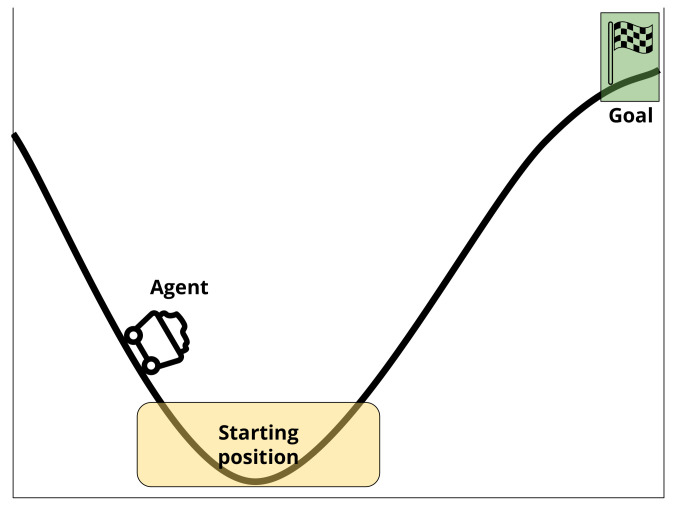
The Mountain Car scenario used in this work. The agent begins at a random position within the starting region shown in yellow and must reach the goal position shown in green. The agent does not have enough power to drive directly to the goal position, therefore, it needs to build up momentum by going back and forth between the hills.

The Mountain Car problem has been chosen for its popularity as an RL testing environment, and because a complete solution can be represented as a set of rules, making the creation of a completely accurate simulated user simple. A complete solution for a problem is not a requirement of a simulated user. The absence of a complete solution simply limits the extent to which certain tests can be performed, such as knowledge level. The Mountain Car problem involves a car, starting in a random location at the bottom of a valley between two hills [1]. The objective is for the car, controlled by the RL agent, to get up one hill to a destination point at the top. This problem is complex as the car does not have enough power to drive directly up a hill. Instead, it must go back and forth between the hills to build up enough momentum to reach the top. Figure 3 illustrates the Mountain Car scenario used in this work.

The agent’s state *s* consists of two state variables, position and velocity, which are represented as real numbers. The position p∈[−1.2,0.6] is the agent’s position within the environment, and v∈[−0.07,0.07] is the velocity of the agent. A velocity greater than zero indicates the agent is travelling to the right or increasing its position. In this scenario, 2 actions are possible, accelerating the car either to the left or the right. At each step, the agent receives a reward *R* of −1, and no reward when reaching the goal state, as shown in Equation (1). The agent was given a learning rate of 0.25, a discounting of 0.9, and used an ϵ-greedy action selection strategy with ϵ=0.05.

(1) $$R\left(s_{t}\right)=\left\{\begin{array}{cc} 0 & \mathrm{if}\;s_{t}\;\mathrm{is}\;\mathrm{goal}\;\mathrm{state}\\ -1 & \mathrm{otherwise} \end{array}\right.$$

To create the simulated user for this experiment three pieces of information are considered: a model of the information the user will provide, a method for altering the accuracy of the advice, and a method for altering the availability of the user. The simulated user requires a model containing at least partial information about the environment or policy so it can automatically evaluate or assist the agent.

For the Mountain Car problem, a complete solution is known and can be used to create a model for the simulated user. For the following Mountain Car experiment, the simulated user employs a heuristic model with a set of rules. The simulated user will agree with the agent if the agent took an action that would accelerate it in its current direction of travel, otherwise, the user disagrees. The rule used to generate the knowledge base for the simulated users is *“agree with the action that accelerates the car in the direction of current velocity”*.

Therefore, the agent used for the experiment is a Boolean-evaluated IntRL agent [51]. The simulated user assists the agent by assessing the agent’s previous action. If the simulated user agrees with the performed action $a_{t}$, a reward shaping signal *S* of +1 is given, as shown in Equation (2). If it disagrees, then the inverse is given, that is, S=−1. If the simulated user has no advice to give, then no additional reward is given.

(2) $$S\left(s_{t}\right)=\left\{\begin{array}{cc} +1 & \mathrm{if}\;v_{t}>0\wedge a_{t}=\mathrm{right}\\ -1 & \mathrm{if}\;v_{t}>0\wedge a_{t}=\mathrm{left}\\ -1 & \mathrm{if}\;v_{t}<0\wedge a_{t}=\mathrm{right}\\ +1 & \mathrm{if}\;v_{t}<0\wedge a_{t}=\mathrm{left}. \end{array}\right.$$

In this set-up, the simulated user is replicating two characteristics of the human-sourced information, accuracy and availability. Both characteristics are represented as percentages. When accuracy is at 100%, the simulated user provides completely accurate advice, and as accuracy decreases the simulated user has an increasing chance of providing incorrect advice. Similarly, when availability is at 100% the simulated user has the opportunity to assess the agent at every time step, and as availability decreases the user’s opportunities to provide advice decreases also. As aforementioned, for the Mountain Car experiment, the simulated user employs a heuristic model with a set of rules. The simulated user will agree with the agent if the agent took an action that would accelerate it in its current direction of travel, otherwise, the user disagrees.

A series of simulated users are created, each with incrementing levels of advice accuracy and availability ranging from 0% to 100%. In total, 36 users are generated, each characteristic incremented by 20%. Although in this illustrative example we include only accuracy and availability, a simulated user may include other human characteristics. For instance, the knowledge level may be implemented as limiting the area in which the simulated user can provide advice, that is, a user with partial knowledge of the environment. Concept drift can be simulated as in multi-objective RL approaches [52] as the relative importance of an objective may be modified as the agent and user work towards a different goal. Reward bias [53,54] can be introduced using biased probability distribution in order to simulate users preferring delivering positive or negative feedback. In the case of cognitive bias, this can be implemented using partially trained agents as advisors [16], for example, an agent performing a task with equivalent parallel paths toward the goal may have a preconceived preference to reach the goal if its knowledge is not optimal, this can be implemented by biasing the Q-values of the trainer agent. Finally, latency can be implemented by simply including a probability of delaying the reward.

The time required to create the proposed simulated users is minimal as only two variables need to be changed. 100 experiments are performed for each of the users, an amount that would not be possible if actual humans were used. The average number of steps the agent takes to complete the mountain climbing task is collected during learning. The agent is given a maximum of 1000 steps each episode to achieve its goal, and the agent is given 100 episodes to learn the task. The experiments produce results showing insights into how the accuracy and availability of the simulated user alter the performance of the IntRL agent.

**Figure 4:**
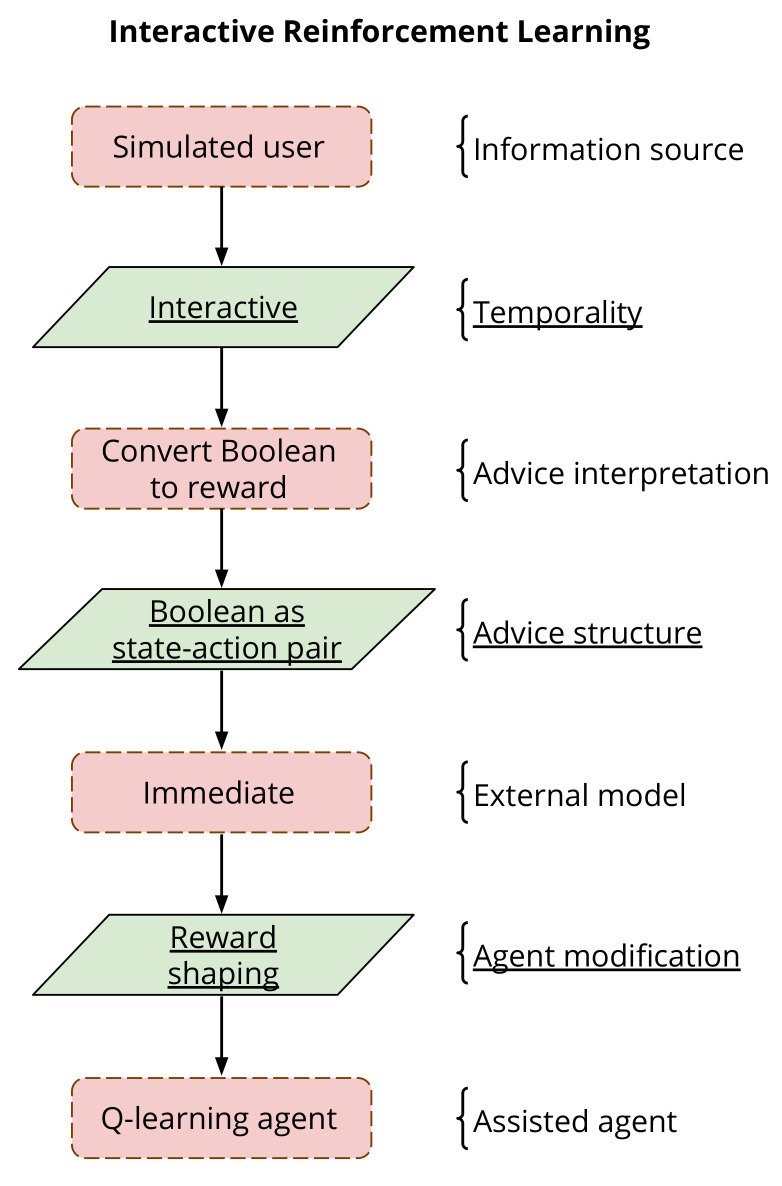
Definition of the evaluative interactive reinforcement learning approach using the assisted reinforcement learning framework [8]. Processing components are displayed in red squares and communication links in green parallelograms.

The IntRL approach proposed in this paper is framed within the assisted reinforcement learning (ARL) taxonomy [8], which proposes a hierarchical framework including processing components and communication links. Figure 4 shows how each processing component and communication link of the proposed IntRL approach is adapted within the ARL taxonomy. Processing components are shown using red rectangles and communication links using green parallelograms with underlined text.

Although previous works have already employed simulated users in other contexts such as spoken dialogue systems [49] or video games [55], they have been employed in an unstructured manner. For instance, in [16] an artificial parent-like trainer agent (with cognitive bias) is proposed as a simulated user in order to train other learning agents during the apprenticeship process in a domestic task. In this experimental setup, different probabilities and consistencies of feedback are tested (equivalent to availability and accuracy respectively). The simulated users in this work are proposed with no structure, this is a common issue that we have observed in previous works employing simulated users [49,55]. In this regard, our methodology may fit with previous work definitions. In the example using a parent-like trainer agent, with our methodology, the representation of human-sourced information would be a stochastic model, the definition of interactive components and links would be state-action par advice with an immediate model and policy-shaping, and the alteration of the human characteristics would be the accuracy, availability, and the cognitive bias.

### 5.2. Results

**Figure 5:**
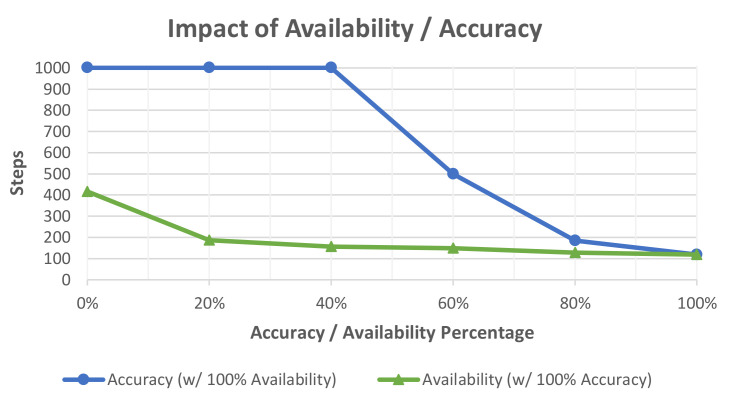
Evaluation of interactive reinforcement learning agents using simulated users with varying levels of availability and accuracy. Each characteristic is incremented by 20% for each experiment with the opposite characteristics set to the maximum. A total of 100 agents are run in each experiment and the steps needed to complete the task are recorded and averaged.

An example of the evaluation that can be performed using this methodology is shown in Figure 5. In this diagram, accuracy and availability of advice is plotted, with the opposite characteristic set to maximum. This graph shows how the agent’s online performance is affected by the accuracy of the advice given by the simulated user. In this case, agent performance quickly degrades as the accuracy of the advice worsens. The largest impact of performance occurs when accuracy falls to 40%, at this point the user is giving incorrect advice more than half of the time. As aforementioned, this experiment uses a Boolean-evaluated IntRL agent. This methodology does not provide a method for the agent to distinguish between human-generated rewards and environmental rewards. This provides a straightforward way for the human to alter the agent’s learning and is responsible for the significant impact shown in Figure 5. When accuracy is high, regardless of advice availability (lower availability indicates few human interactions), the performance of the agent is greatly improved. However, when accuracy is low, performance quickly decreases with no method for recovery if inaccurate human evaluations continue. The figure also shows that advice availability, when 100% accurate, has a very large impact on agent performance, but the rate of change is diminishing as availability increases.

**Figure 6:**
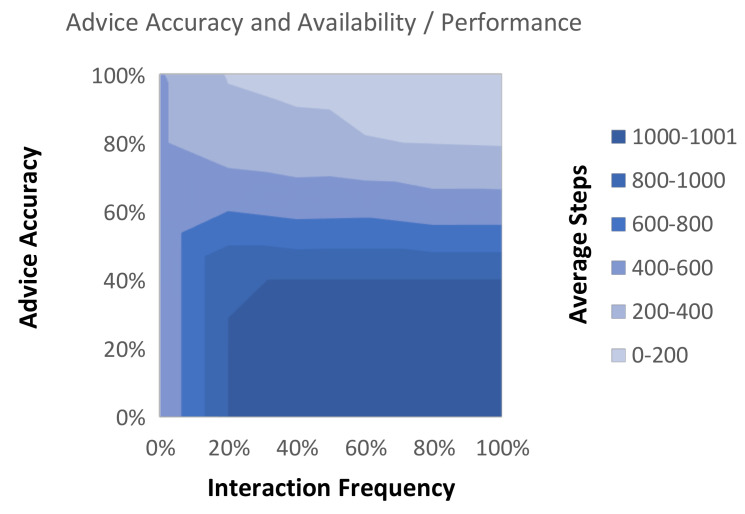
Impact of accuracy and availability on the agent performance. The interactive reinforcement learning agent uses a simulated user with different levels of availability and accuracy. For each characteristic, an increment of 20% is set for each experiment and the total average steps are shown.

The contour graph shown in Figure 6 is a method for presenting the relationship between two characteristics of human-sourced information and their effect on the performance of the agent. In Figure 6 the average steps of the agent are plotted, showing the change in performance which is observed as the simulated users’ accuracy and availability are altered. From this figure, some observations can be made regarding how the accuracy and availability of the user impact the average performance of the agent. For example, it can be observed that just a small amount of advice can have a large impact on the agent’s performance; however, there are diminishing returns as frequency increases. The figure also shows that inaccuracy of advice has less of an impact as the frequency of advice is increased. From these observations, it may be concluded that a small amount of largely accurate advice is enough to greatly accelerate agent learning.

These methods of evaluation allow for greater insight into the performance of IntRL agents when advised by different users. The use of different simulated users can show how an IntRL agent can perform under various conditions. The application of simulated users for this method of evaluation can identify weaknesses and strengths with an IntRL agent and the user providing advice, while also performing the experiments much more cheaply and faster than actual humans.

## 6. Conclusions

In this work, we introduced a general methodology for evaluating interactive reinforcement learning by employing simulated users as a substitute for actual humans. While simulated users are not a replacement for real human testing, it was demonstrated that evaluations using simulated users could show detailed insights into how the agent is expected to act under certain interaction conditions. A series of interaction characteristics were introduced that may impact the quality and applicability of the information humans provide as well as principles of evaluation of simulated users.

Some of these characteristics were replicated in an illustrative experiment, showing how such characteristics impact agent performance and how the results gained from simulated users can provide indicative observations on real human performance. The experiment carried out acts as a proof-of-concept for more detailed evaluations in the future. However, additional experiments are necessary in order to properly compare simulated users as substitutes for human advisors in more complex real-world scenarios. An important aspect to evaluate is the time needed for setting up the simulated user. This may be a crucial factor in order to effectively use the proposed methodology, otherwise, the effort may not be worth it. There are opportunities for further research into the applicability of simulated users for evaluation purposes and how to best represent the variety of interactions that may occur. The methodology and characteristics introduced here can help to make the development of interactive reinforcement learning agents an increasingly viable option for machine learning.

## Data Availability

Data is contained within the article.
